# Hyperphosphorylated Tau Targeting Human Serum Albumin Fusion Protein as Therapeutics for Alzheimer’s Disease

**DOI:** 10.1002/alz.086545

**Published:** 2025-01-09

**Authors:** Sookhee Bang, Jeong Keun Song, Kwan Hee Lee

**Affiliations:** ^1^ L & J Bio, Co., Ltd, Seoul, Songpa‐Gu Korea, Republic of (South)

## Abstract

**Background:**

Neurofibrillary tangles (NFTs), along with amyloid beta plaque, are neuropathological aggregates of Alzheimer’s Disease (AD). Hyperphosphorylated tau is responsible for the NFTs formation and further neurodegeneration in AD. The hippocampal region and the entorhinal cortex (EC) have been a major focus of AD research because the deposits of hyperphosphorylated tau protein and NFT in these regions are correlated with memory deficits. The AD mouse model of human microtubule‐associated protein tau (MAPT) P301 mutations is a reflective model of tau phosphorylation, a key driver of NFTs seen in human AD. AL04 is a recently developed recombinant fusion protein which consists of Cystatin C, human serum albumin and the novel blood brain barrier (BBB) penetrating peptide. In our previous paper, AL04 was reported to reduce amyloid beta plaques in the Tg2576 mouse which is the amyloid beta AD model.

**Method:**

In this study, we investigated the effect of AL04 on lowering hyperphosphorylated tau in JNPL3 mice carrying pathogenic human tau P301L mutation. We injected AL04 (5mg/kg) or PBS intraperitoneally to 3‐month‐old JNPL3 mice every other week for 24 weeks, respectively. We used confocal microscopy and western blot analysis to visualize the changes in hyperphosphorylated tau Ser202/Thr205 labeled with AT8 antibody in the hippocampus and EC.

**Result:**

We found that the AL04 treatment decreases the hyperphosphorylated tau and NFTs in the hippocampus and EC. We observed the reduction of I2PP2A (inhibitor of Protein Phosphatase 2A, PP2A) and increased the expression levels of autophagy regulating proteins in the brain homogenates of AL04 treated mice.

**Conclusion:**

Our data suggested that AL04 can be a prophylactic/therapeutic agent for AD because it can reduce the hyperphosphorylated tau and NFTs. In combination of our previous research, AL04 can be a dual action therapy to reduce both amyloid beta aggregate and NFTs.

